# Synthesis and Toxicity Evaluation of New Pyrroles Obtained by the Reaction of Activated Alkynes with 1-Methyl-3-(cyanomethyl)benzimidazolium Bromide

**DOI:** 10.3390/molecules26216435

**Published:** 2021-10-25

**Authors:** Beatrice-Cristina Ivan, Florea Dumitrascu, Adriana Iuliana Anghel, Robert Viorel Ancuceanu, Sergiu Shova, Denisa Dumitrescu, Constantin Draghici, Octavian Tudorel Olaru, George Mihai Nitulescu, Mihaela Dinu, Stefania-Felicia Barbuceanu

**Affiliations:** 1 Faculty of Pharmacy, “Carol Davila” University of Medicine and Pharmacy, 6 Traian Vuia Street, 020956 Bucharest, Romania; cristina.ivan@drd.umfcd.ro (B.-C.I.); adriana.anghel@umfcd.ro (A.I.A.); robert.ancuceanu@umfcd.ro (R.V.A.); george.nitulescu@umfcd.ro (G.M.N.); mihaela.dinu@umfcd.ro (M.D.); stefania.barbuceanu@umfcd.ro (S.-F.B.); 2 “Costin D. Nenitescu” Center of Organic Chemistry, Romanian Academy, 202B Splaiul Independenței, 060023 Bucharest, Romania; cst.drag@yahoo.com; 3 Laboratory of Inorganic Polymers, “Petru Poni” Institute of Macromolecular Chemistry, 41A Aleea Grigore Ghica Voda, 700487 Iasi, Romania; shova@icmpp.ro; 4 Faculty of Pharmacy, “Ovidius” University Constanta, Cpt. Av. Al. Serbanescu Street, 900470 Constanta, Romania; denisa.dumitrescu2014@gmail.com

**Keywords:** benzimidazolium bromide, alkynes, 1,3-dipolar cycloaddition, pyrroles, X-ray diffraction, toxicity evaluation

## Abstract

A series of new pyrrole derivatives were designed as chemical analogs of the 1,4-dihydropyridines drugs in order to develop future new calcium channel blockers. The new tri- and tetra-substituted *N*-arylpyrroles were synthesized by the one-pot reaction of 1-methyl-3-cyanomethyl benzimidazolium bromide with substituted alkynes having at least one electron-withdrawing substituent, in 1,2-epoxybutane, acting both as the solvent and reagent to generate the corresponding benzimidazolium N3-ylide. The structural characterization of the new substituted pyrroles was based on IR, NMR spectroscopy as well as on single crystal X-ray analysis. The toxicity of the new compounds was assessed on the plant cell using *Triticum aestivum* L. species and on the animal cell using *Artemia franciscana* Kellogg and *Daphnia magna* Straus crustaceans. The compounds showed minimal phytotoxicity on *Triticum* rootlets and virtually no acute toxicity on *Artemia* nauplii, while on *Daphnia magna*, it induced moderate to high toxicity, similar to nifedipine. Our research indicates that the newly synthetized pyrrole derivatives are promising molecules with biological activity and low acute toxicity.

## 1. Introduction

**Figure 1:**
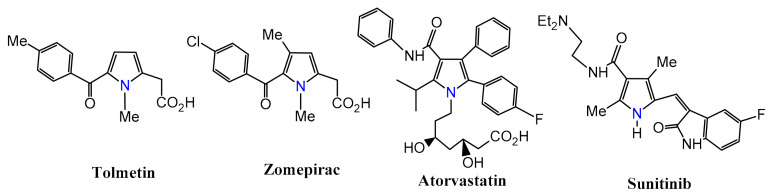
Structure of some pyrrole drugs.

**Figure 2:**
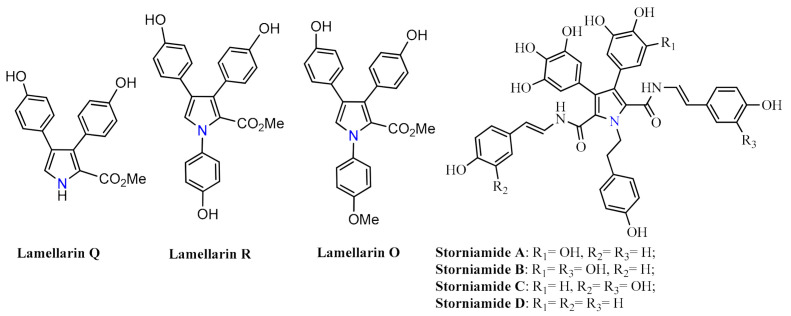
Structure of some marine pyrrole alkaloids.

Pyrrole is a five membered heteroaromatic compound with one nitrogen atom in the ring. The pyrrole core is usually encountered among natural compounds, the best-known being the heme, chlorophyll, vitamin B12, and marine alkaloids (e.g., lamellarins, storniamides) [1,2,3,4,5,6]. The importance of natural and synthetic pyrroles arises from their wide range of applications in various fields such as medicinal chemistry, catalysis, dyes, and materials science [5,6,7,8,9,10,11,12,13]. A great number of natural or synthetic pyrrole derivatives have been found to exhibit a variety of biological activities such as antimicrobial, anti-inflammatory, anticancer, antiviral, antihypertensive, etc. [2,5,6,7,8,9,10,11,14,15,16]. The best-known marketed drugs containing substituted pyrrole nucleus [6,7,8,10] are tolmetin and zomepirac, nonsteroidal anti-inflammatory drugs used for treatment of rheumatoid arthritis and pain [10]; atorvastatin used for the treatment of dyslipidemia and to prevent cardiovascular diseases [7]; and sunitinib used in cancer treatment [17] (Figure 1). From the marine alkaloid group with the tri- and tetrasubstituted pyrrole ring, lamellarins O, Q, R for their anticancer activity [5] and storniamids A–D for antibacterial activity [3] stand out (Figure 2). Additionally, pyrroles are useful building blocks [18,19,20,21] in the design and synthesis of naturally occurring compounds, functionalized materials, and new bioactive condensed heterocycles, and as a consequence, the interesting synthetic aspects and applications of the compounds possessing the pyrrole core have been reviewed [9,10,11,12,13,18,22,23,24,25,26,27,28,29].

**Figure 3:**
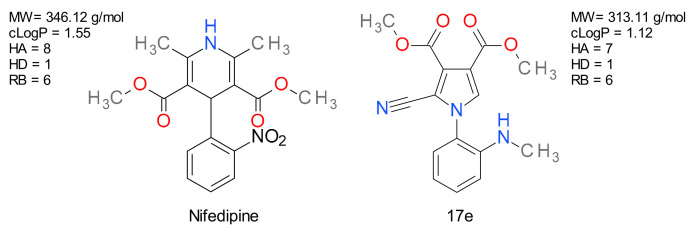
The structure of nifedipine and of the new pyrrole compound **17e** together with relevant structural descriptors (MW = molecular weight, cLogP = logarithm of the octanol/water partition coefficient, HA = hydrogen bond acceptors count, HD = hydrogen bond donors count, RB = rotatable bonds count).

The 1,4-dihydropyridine (DHP) class is frequently the first choice of drug used in the treatment of hypertension [30] and angina [31]. Their mechanism of action is the inhibition of the L-type calcium-dependent calcium channel in the cardiovascular system. The new synthetized compounds were designed to resemble the structure of nifedipine, the prototype for the DHP class, by using a phenyl substitution at the *N*-pyrrole atom and a methlyamino group as substitution for the nitro group of nifedipine. In Figure 3, the structure of one of the new compounds (**17e**) is presented next to that of nifedipine together with relevant structural descriptors.

Herein, we present the synthesis of new highly substituted pyrroles in a two stage process involving the formation of 1-methyl-3-(cyanomethyl) benzimidazolium bromide and its subsequent reaction with acetylenic dipolarophiles in 1,2-epoxybutane in the second step. The prediction of molecular mechanism of action and toxicity was used along with the in vitro evaluation of toxicity against plant cells (*Triticum aestivum* L.) and animal organisms, *Artemia franciscana* Kellogg and *Daphnia magna* Straus.

## 2. Results and Discussions

### 2.1. Chemistry

**Scheme 1:**
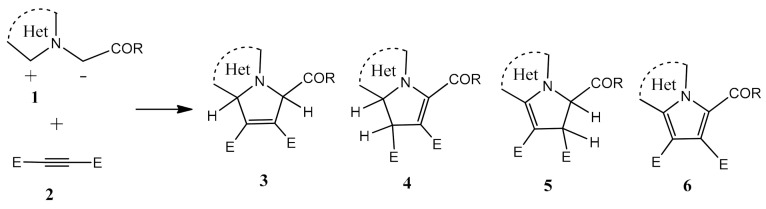
General synthesis of pyrrolo-fused heteroaromatic compounds via 1,3-dipolar cycloaddition reaction.

The 1,3-dipolar cycloaddition reaction between heteroaromatic *N*-ylides **1** and acetylenic dipolarophiles **2** (Scheme 1) is one of the most accessible synthetic procedures in the construction of condensed aromatic *N*-heterocycles of type **6** possessing a pyrrole ring [32,33,34,35,36,37,38,39,40,41,42]. The final reaction products **6** result via the initial formation of the dihydroderivative structure **3**, followed by its rearrangement to **4** or **5** (Scheme 1). In the reaction conditions or in the presence of an oxidizing reagent, these intermediates undergo further aromatization to the pyrrolo-condensed compounds **6**.

The isolation and characterization of dihydroderivatives of types **3**–**5** were reported in several cases [32,37,38,39]. Usually, the heteroaromatic *N*-ylides **1** are generated in situ by the action of a base on the starting cycloimminium salts, which were previously obtained in mild reaction conditions by the condensation between *N*-aromatic heterocycles and activated halogenated derivatives.

**Scheme 2:**
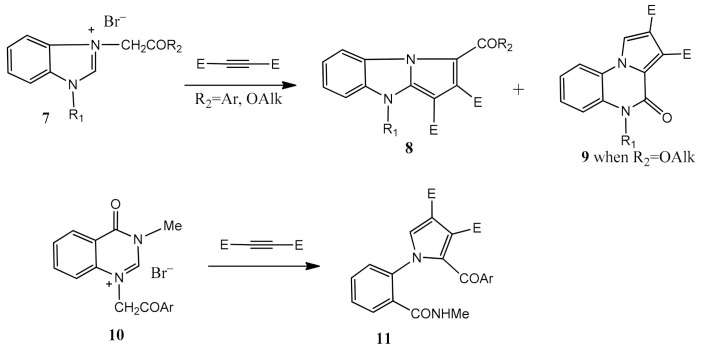
Polysubstituted pyrrole structures starting from different cycloimmonium structures.

Interestingly, the reaction of benzimidazolium salts **7** with activated alkynes (Scheme 2) in the presence of bases gives the expected pyrrolo[1,2-*a*]benzimidazoles **8** along with pyrrolo[1,2-*a*]quinoxalines **9** [41]. Moreover, the nature of the substituent attached at the nitrogen can direct the course of this reaction toward substituted pyrroles [35]. The formation of highly substituted pyrroles **11** from N3-quinazolinonium bromides **10** and activated alkynes was also reported (Scheme 2) [32]. The dihydropyrolobenzimidazoles, intermediates resulting from the cycloaddition between activated benzimidazolium ylide and acetylene, can undergo two types of reactions. A first pathway consists in the dehydrogenation to pyrrolobenzimidazoles **8**, a common pathway for most cycloadducts. The other way is to open the imidazole ring with the formation of an intermediate pyrrole. Depending on the nature of the radical R_2_, the intermediate pyrroles can be isolated or cyclized to pyrroloquinoxaline **9** derivatives [32,35].

In the case when bromoacetonitrile is used as an alkylating agent to the N3 of the benzimidazole ring, the [3 + 2] cycloaddition between the corresponding benzimidazolium *N*-ylide and ethyl 2,2-dihydropoly-fluoroalkanoates led to the formation of substituted 1-arylpyrroles as a majority product. The reaction mechanism involves the formation of the dihydropyrrolebenzimidazole derivative, followed by the opening of the imidazole ring [36].

**Scheme 3:**
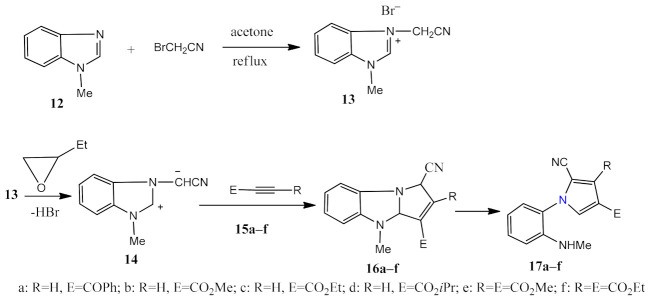
Highly efficient synthesis of new polysubstituted pyrroles via 1,3 dipolar cycloaddition of benzimidazolium *N*-ylide **14**.

The synthesis of the new highly substituted pyrroles involves commercially available materials and a two-stage procedure. In the first step, 1-methyl-3-cyanomethylbenzimidazolium bromide **13** [36] is easily prepared in good yield by N3-alkylation of 1-methylbenzimidazole **12** with bromoacetonitrile, in acetone, under reflux (Scheme 3). The structure of the benzimidazolium bromide **13** was confirmed by NMR and IR spectroscopy. The methylene group from benzimidazolium bromide **13** was highlighted in the ^1^H-NMR spectrum by the singlet signal, which appears at 6.03 ppm, and in the ^13^C-NMR spectrum at 34.9 ppm. Additionally, the CN signal from 114.3 ppm confirms that the reaction of 1-methylbenzimidazole **12** with bromoacetonitrile has taken place. Noteworthy, in the IR spectrum of benzimidazolium bromide, the peak intensity corresponding to the wave number of CN group is very weak.

In the second step, the synthesis of new pyrroles **17a**–**f** was achieved by reaction between 1-methyl-3-cyanomethylbenzimidazolium bromide **13** with acetylenic dipolarophiles **15a**–**f** in 1,2-epoxybutane under reflux. After working up the reaction mixture, the new pyrroles **17a**–**f** were isolated in moderate yields.

The reaction mechanism for the synthesis of new substituted pyrroles **17a**–**f** from **13** implies the generation of benzimidazolium ylide **14** under action of 1,2-epoxybutane followed by its [3 + 2] cycloaddition with acetylene reactants **15a**–**f** to form the unstable pyrrole[1,2-a]benzimidazole primary adducts **16a**–**f**. Under reaction conditions, these intermediates **16** undergo imidazole ring opening giving substituted pyrroles **17a**–**f** (Scheme 3).

The structure of the new pyrroles **17** was confirmed by IR and NMR spectroscopy and by single crystal X-ray diffraction analysis for compound **17c**. Good evidence for the formation of pyrroles is the presence in their IR spectra of a stretching band in the range 3341–3421 cm^−1^ corresponding to NH groups. The absorption bands in the region 2220–2232 cm^−1^ were attributed to the stretching vibrations of C≡N groups. The carbonyl group bands from the keto pyrrole **17a** and pyrrole esters **17b**–**f** could be observed in the range 1637–1752 cm^−1^. The main feature of ^1^H-NMR spectra that confirms the pyrrole structure of compound **17a**–**f** is the presence of the NH group, which in the case of pyrroles **17a**–**c** registered in CDCl_3_ appears as a quartet (3.42–3.55 ppm) due to the coupling with the methyl group. Pyrroles **17d**–**f** were recorded in similar conditions, but the coupling between NH and Me in the MeNH group was not observed, being present as a broad singlet signal (3.38–3.42 ppm). For trisubstituted pyrroles **17a–d**, those two protons from the pyrrole ring appeared as two doublets (7.29–7.45 ppm for H-3 and 7.45–7.51 ppm for H-5) with a coupling constant of 1.6 Hz, whereas for tetrasubstituted pyrroles **17e**,**f**, the proton from the pyrrole ring (H-5) appears as a sharp singlet at 7.36 ppm.

In the ^13^C-NMR spectra, the characteristic signals for pyrrole moiety are those of C-3 (~122 ppm) and C-5 (~132 ppm), the latter being strongly deshielded due to its vicinity to the nitrogen atom from the pyrrole ring. The signal of C=O carbon from the benzoyl group (**17a**) appears at 188.4 ppm and from the ester group resonates in the range of 161.4–163.3 ppm. The CN carbon signal was observed at δ = 110.5–112 ppm. All the carbon atoms in the aryl and alkyl groups appeared at the expected chemical shifts.

**Figure 4:**
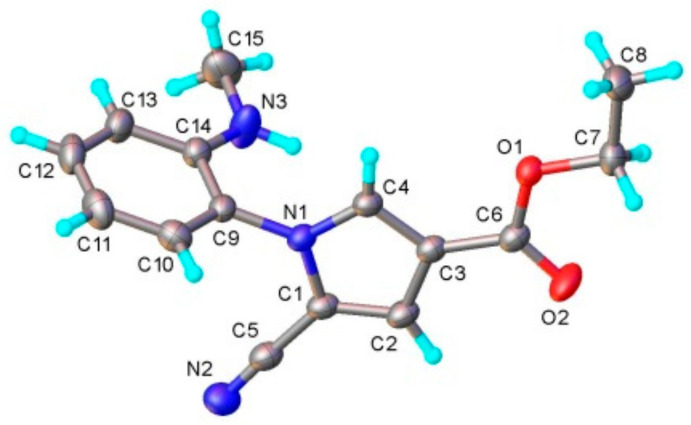
X-ray molecular structure of **17c** with atom labeling and thermal ellipsoids at the 50% level.

**Figure 5:**
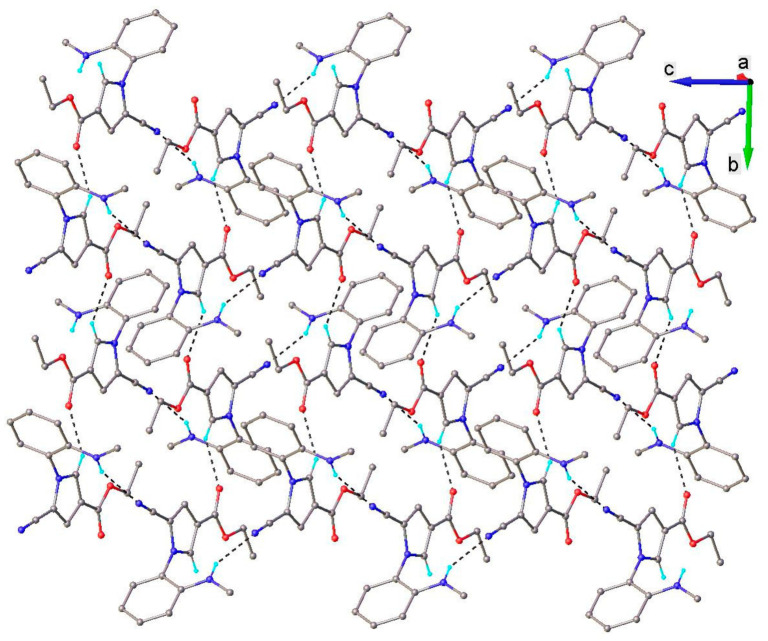
2D-supramolecular network in the crystal structure **17c**. Non-relevant are omitted. H-bonds parameters: N3-H⋅⋅⋅N2 [N3-H 0.88 Å, H⋅⋅⋅N2 2.30 Å, N3⋅⋅⋅N2(*x*, 0.5–*y*, *z*–0.5) 3.075(2) Å, ∠N3HN2 147.4°]; C4-H⋅⋅⋅O2 [C4-H 0.95 Å, H⋅⋅⋅O2 2.36 Å, C4⋅⋅⋅O2(1–*x*, 0.5 + *y*, 0.5–*z*) 3.075(2) Å, ∠C4HO2 143.7°].

**Figure 6:**
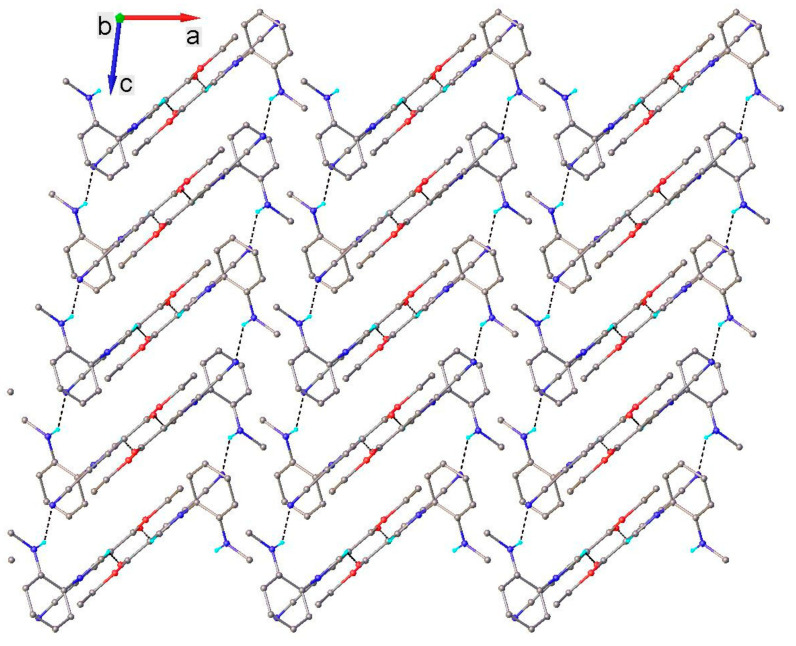
Crystal structure packing viewed along the b-axis. Non-relevant H-atoms are omitted.

The structures of the pyrroles were confirmed by single crystal X-ray diffraction study of the representative pyrrole **17c**. This compound crystallizes in the P21/c space group of the monoclinic system, its molecular structure being constructed from neutral units, as shown in Figure 4. The **17c** molecule is essentially non-planar: the dihedral angle between two planar fragments formed by pyrrole and phenyl rings (along with their substitutes) was 96.523(2)°. The analysis of the molecular structure shows the presence of atom groups that can serve as donors and acceptors for intermolecular H-bonding. As a result, the neutral molecules are interacting in the crystal to form two-dimensional supramolecular layers, as illustrated in Figure 5. The crystal structure is generated via parallel packing of the above-mentioned, weakly interacting, 2D supramolecular architectures. A partial crystal packing viewed along the b-axis is shown in Figure 6. The crystallographic and refinement details are given in Table 1.

### 2.2. Toxicity Evaluation

#### 2.2.1. Plant Toxicity Evaluation

**Figure 7:**
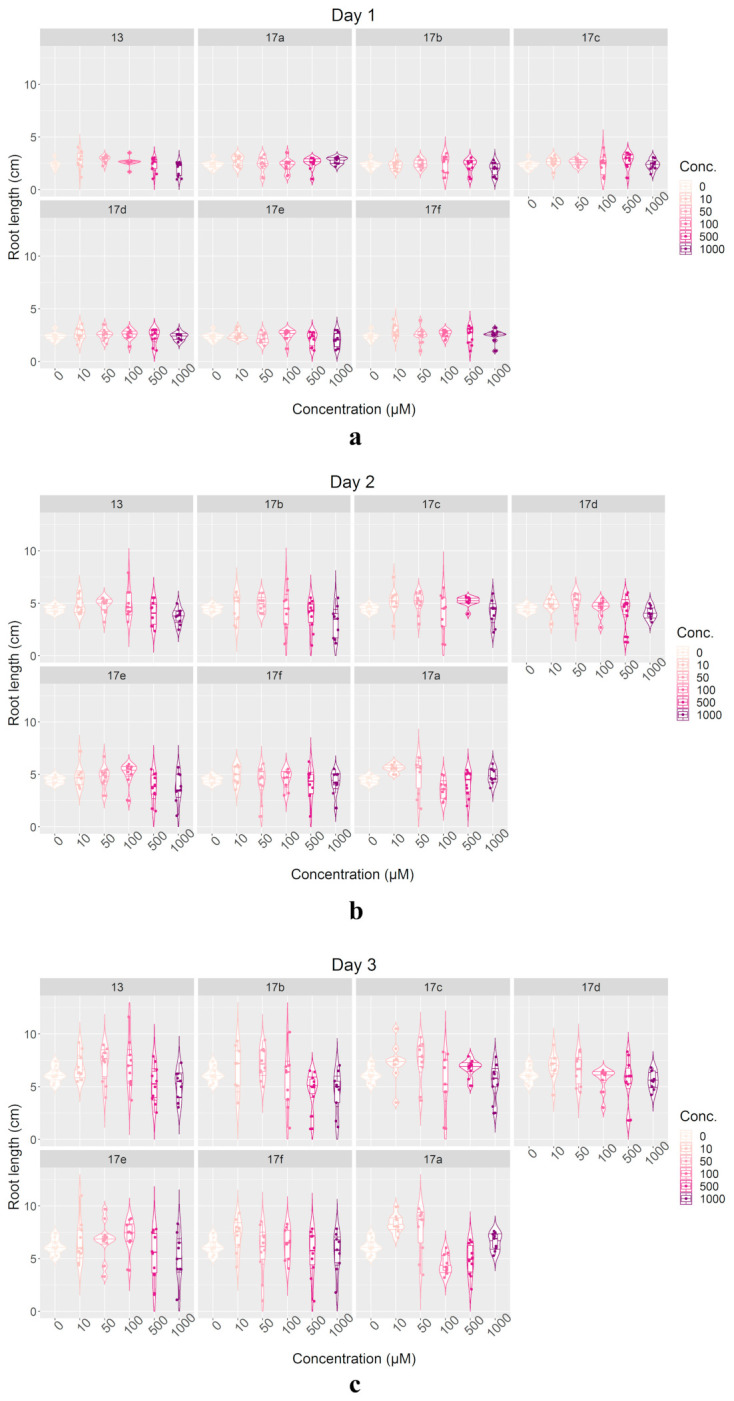
Violin plots (with boxplots inside to indicate quartiles) describing the *Triticum* root length as a function of the tested compounds and concentrations, in: (**a**) day one, (**b**) day two, (**c**) day three of exposure.

Except for compound **17b**, the mixed-effects models based on all data indicated a statistically significant inhibitory effect for the two highest concentrations (*p* < 0.001 and *p* = 0.006, respectively), a significantly stimulatory effect for the two lowest concentrations (*p* < 0.001), and a null effect for the middle concentration (Figure 7). Except for derivative pyrrole **17b**, which exerted an inhibitory effect, the other compounds did not differ significantly from salt **13**. Analyses performed on measurements carried out on the second day were generally consistent with this finding. Ordinary least squares regression as well as the different versions of robust regression (treating the control as the zero concentration level) were similar in their results, indicating very small differences among the tested compounds (**13**, **17a** to **17f**) and a small effect for the concentration: the standardized coefficients were not larger than 0.16 (in absolute value) (i.e., their influence on root growth was not larger than 0.16 standard deviations). The coefficients of determination for the different models were around 0.09, which means that the contribution of substances and concentration to the variability observed in *Triticum* root length were limited to less than 10% (i.e., a very small effect). A very small inhibitory effect was only found for the highest two concentrations (1000 and 500 μM)—standardized coefficients of −0.15 and −0.10 in the OLS model, an almost null effect for the middle concentration (beta −0.01) and a small stimulatory effect at the lowest two concentration levels (standardized coefficients of 0.10 and 0.15, respectively); the effect was significant only for the highest concentration level (1000 μM) and two lowest levels (10 and 50 μM). Using salt **13** as a reference, none of the other compounds differed significantly in their inhibitory effects, the majority rather having slightly (but statistically non-significant at the conventional 0.05 threshold) stimulatory effects, with the possible exceptions of **17c** (*p* = 0.024 in the “lmRob” robust regression model and *p* = 0.06 in a “lmrob” regression) and **17a** (*p* = 0.026 in the “lmRob” model, but *p* = 0.172 in the “lmrob” model). Treating the control group as a substance (as opposed to the zero level concentration), none of the compounds differed significantly from it. Considering the very small effects of the compounds and concentrations, no IC_50_ values are reported, as they would make little sense (and the 95% confidence intervals were very wide). This may allow for the conclusion that the compounds have little phytotoxic effects on *Triticum aestivum* in the experimental setting used.

**Figure 8:**
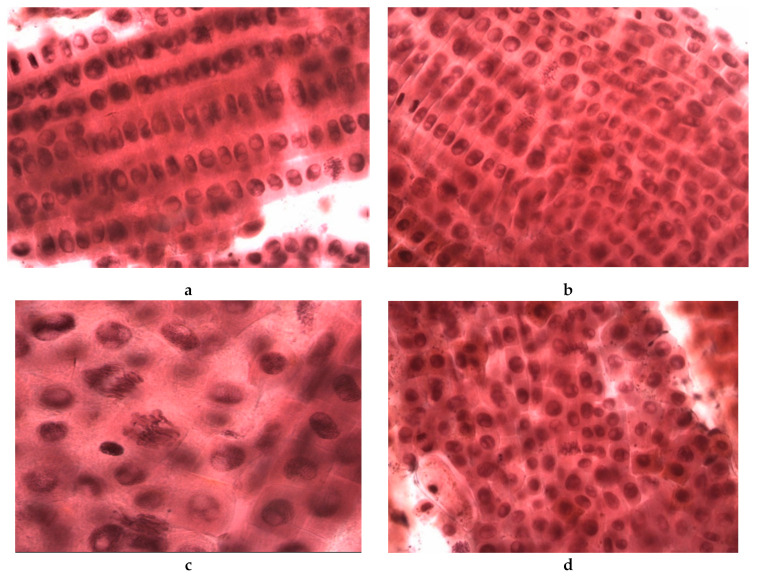
(**a**) Telophase with slight tropokinesis observed in rootlets treated with compound **13** (conc. 1000 µM). (**b**) Normal metaphases, telophases, and slight tropokinesis observed in rootlets treated with compound **17b** (conc. 10 µM). (**c**) Disorganized metaphases or in tropokinesis observed in rootlets treated with compound **17a** (conc. 10 µM). (**d**) Normal telophase and metaphases as well as in tropokinesis observed in rootlets treated with compound **17d** (conc. 100 µM). All photomicrographs were obtained with a 40× objective.

At the microscopic level, the effects of the compounds tested consisted in mitotic changes observed in the rootlet apex cells of *Triticum aestivum* L. These were observed after 24 h from the contact of germinated karyopses with the tested compounds at different concentration levels in comparison to the DMSO control. Changes were minor and consistent with the macroscopic observations. Thus, mitotic phases were seen in all microscopic preparations, besides interphase nuclei. These were mostly normal, but slight tropokinesis, hypertrophied nucleoli, and disorganized metaphases were occasionally observed (Figure 8).

#### 2.2.2. Animal Toxicity Evaluation

##### Artemia Franciscana Toxicity Assay

The lethality of the compounds tested on *Artemia crustaceans* was 0% both in the first 24 h and at 48 h, which implies that the substances were virtually devoid of acute toxicity. This result is also in agreement with the low toxicity observed in the phytotoxicity assessment.

##### Daphnia Magna Toxicity Assay

**Figure 9:**
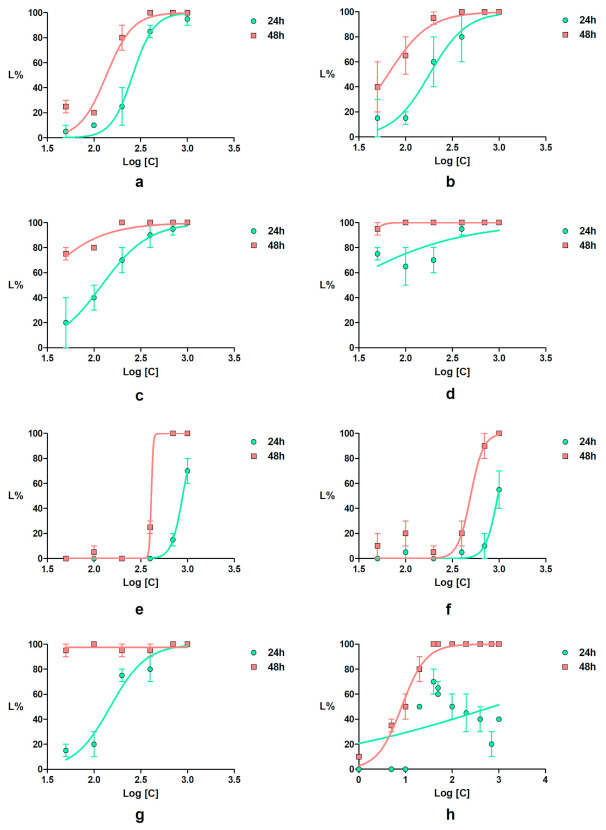
*Daphnia magna* lethality curves for the tested compounds: (**a**)—**13**; (**b**)—**17a**; (**c**)—**17b**; (**d**)—**17c**; (**e**)—**17d**; (**f**)—**17e**; (**g**)—**17f**; (**h**)—nifedipine; error bars represent the standard error of the mean.

The results of the *Daphnia magna* bioassay are presented in Table 2, and the lethality curves in Figure 9. At 24 h, the lethality induced by compounds **17a**, **17b**, **17c**, and **17f** was ≥95% between 700 and 1000 µM, and at 48 h, these compounds induced 100% lethality at most levels of concentration. Because of the higher lethality recorded at 48 h, the LC_50_ values could not be calculated for compounds **17b**, **17c**, and **17f**, their values being lower than 50 µM. Compounds **17d** and **17e** induced lower toxicity at both 24 and 48 h. The lethality of decreasing order of toxicity of newly synthetized compounds at 48 h was: **17b**, **17c**, and **17f** with a LC_50_ lower than 50 µM, followed by **17a**, **17d**, and **17e**. The LC_50_ value of **13** revealed a medium toxicity, being more toxic than **17d** and **17e**, and having a lower toxicity than compounds **17a**, **17b**, **17c**, and **17f**. Nifedipine induced a maximum of 65% lethality at 24 h, and the effect was not correlated with the concentration between 40 and 1000 µM. Our results regarding the nifedipine low toxicity at 24 h are consistent with the results of Tan et al. [43]. At 48 h of exposure, 100% lethality was recorded for all concentrations between 40 and 1000 µM, the mortality being correlated with the concentrations, with a correlation index higher than 0.95. Although for nifedipine, the LC_50_ is not reported in the literature, results regarding the toxicity are available for verapamil, another Ca channel inhibitor that partially shares the mechanism of action [44]. Verapamil’s LC_50_ value was 15.4 µM, which means a 2-fold lower toxicity than nifedipine. Moreover, verapamil caused in *Daphnia magna* heart a concentration-dependent acceleration at concentrations ranging from 0.1 to 10 µM, and adverse reactions such as decreased frequency at 100 µM. The contradictory results of the biological activity evaluation could be explained either by the lack of target receptors or by the presence of certain enzymes that lead to the inactivation of newly synthetized pyrrole derivatives in *Triticum aestivum* and *Artemia franciscana*.

The *Daphnia magna* results showed that compounds **17b**, **17c**, and **17f** generated similar levels of toxicity as nifedipine. Compound **17a** could also be included along with the mentioned compounds, mainly due to its 95% CI.

### 2.3. Prediction of the Molecular Mechanism of Action and Toxicity

#### 2.3.1. PASS Prediction

The software PASS (Institute of Biomedical Chemistry, Version 2.0, Moscow, Russia) (acronym for “prediction of activity spectra for substances”) can predict the potential of a compound to produce a large number of biological activities or to interact with various biological targets. The method used the structure of each given molecule as input data and returned an array of data consisting of the probability of the compound to be active (Pa) or inactive (Pi) [45]. The corresponding Pa values for biological activities related to the calcium channel blockers are presented in Table 3 together with nifedipine as a comparison standard.

With the exception of **17a**, all the new pyrrole derivatives presented low but significant Pa values to function as calcium channel blockers. These results may be evidence of the importance of the presence of an ester function. Even so, compound **17a** is predicted to produce some effects that are characteristic of the calcium channel antagonists such as the anti-ischemic effect.

There were small differences between the Pa values obtained for the compounds **17b**–**f**. The compound **17d** has the highest chances to block the calcium channels and to produce antihypertensive, anti-anginal, and anti-ischemic effects.

**Figure 10:**
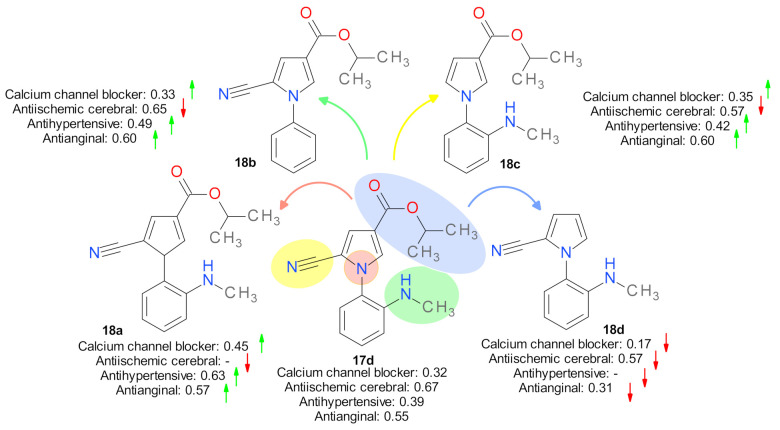
The PASS predicted Pa values after simple transformations applied to compound **17d**. The arrows indicate the rapport of each Pa value with that corresponding to compound **17d**.

In order to evaluate the role of each functional group, compound **17d** was chosen as a starting point because it returned the highest Pa values on antihypertensive, anti-anginal, and anti-ischemic effects. Four structures were drawn by simple transformations of the **17d** structure and a PASS analysis was performed on each one. The results are presented in Figure 10.

The elimination of the ester group is predicted to reduce the probability of structure **18d** to have antihypertensive, anti-anginal, and cerebral anti-ischemic effects indicating the importance of this element. The removal of the nitrile or the methylamino groups changes the probability of blocking the calcium channels very little, and slightly improves the potential of antihypertensive and anti-anginal effects. Interestingly, the substitution of the pyrrole nitrogen with a carbon atom is predicted to improve the chances to function as a calcium channel blocker and to produce antihypertensive and anti-anginal effects. However, this transformation eliminates the cerebral anti-ischemic properties.

The PASS analysis demonstrated the hypothesis of the drug design strategy and also revealed an interesting new potential pharmacological activity, the anti-arthritic effect. Based on this observation, the results were analyzed in order to find related activities. The Pa values are presented in Table 4.

The Pa values indicate that the anti-arthritic effect of compounds **17a**–**d** was correlated with the potential to inhibit the interleukin activity, especially that of interleukin 2. Compounds **17e** and **17f** are predicted not to have these effects, probably because they possess an additional ester group.

#### 2.3.2. Predicted Acute Rat Toxicity

The predicted median lethal dose (LD_50_) of the new compounds after oral and intravenous (IV) administration on rats are presented in Table 5 and indicate a relatively low degree of toxicity. For some compounds, the predicted oral LD_50_ values fell out of the applicability domain (AD) of the application, but all of the IV LD_50_ were inside the AD.

Nifedipine was used as a comparison standard and its predicted oral LD_50_ of 1225 mg/kg was very close to that reported by the literature of 1022 mg/kg [46].

## 3. Materials and Methods

### 3.1. Chemistry

The melting points were measured using a Boetius hot plate microscope (Carl Zeiss, Jena, Germany) and are uncorrected. The ^1^H-NMR and ^13^C-NMR spectra were recorded on a Varian Gemini 300BB spectrometer (Varian, Palo Alto, CA, USA) operating at 300 MHz for ^1^H and 75 MHz for ^13^C. The spectra were recorded in deuterated solvents, CDCl_3_ or DMSO-d_6_, at 298 K and the chemical shifts δ are in parts per million (ppm) relative to TMS used as the internal standard. The coupling constants values *J* are reported in hertz (Hz) and the signals’ multiplicity are abbreviated as follows: s, singlet; d, doublet; dd, doublet of doublets; t, triplet; q, quartet; spt, septet; m, multiplet; and b, broad. The IR spectra were registered on a Fourier transform (FT)IR Vertex 70 spectrometer (Bruker Optik GmbH, Ettlingen, Germany) in ATR modes. The elemental analysis was performed on a Costech Instruments EAS 32 apparatus (Costech Analytical Technologies, Valencia, CA, USA) and the results were in agreement with the calculated values. All starting materials and solvents were purchased from common commercial suppliers and were used without further purification.

The X-ray diffraction measurements were carried out with an Oxford-Diffraction XCALIBUR E CCD diffractometer (Rigaku Oxford Diffraction, Sevenoaks, Kent, UK) equipped with graphite-monochromated MoKα radiation. Single crystals were positioned at 40 mm from the detector and 288 frames were measured each for 5 s over 1° oscillation width. The unit cell determination and data integration were carried out using the CrysAlis package of Oxford [47]. The structure was solved by direct methods using Olex2 [48] software (Rigaku Corporation, Version 1.171.38.46, Oxford, UK) with the SHELXS structure solution program and refined by full-matrix least-squares on F^2^ with SHELXL-2015 [49] using an anisotropic model for non-hydrogen atoms. All H atoms attached to carbon were introduced in idealized positions (d_CH_ = 0.96 Å) using the riding model with their isotropic displacement parameters fixed at 120% of their riding atom (CCDC 2007999).

#### 3.1.1. Synthesis of 1-Methyl-3-cyanomethyl-benzimidazolium Bromide 13

1-Methylbenzimidazole 12 (1.33 g, 10 mmol) and bromoacetonitrile (15 mmol, 1 mL) were dissolved in 100 mL acetone and the reaction mixture was refluxed for 10 h. After cooling, the precipitate was filtered and washed with acetone on the filter and used in the next step without purification. The compound was obtained as colorless crystals in 87% yield. ^1^H-NMR (300 MHz, DMSO-d_6_) δ ppm: 4.13 (s, 3H, Me), 6.03 (s, 2H, CH_2_), 7.78–8.18 (m, 4H, H-4, H-5, H-6, H-7), 9.89 (s, 1H, H-2). ^13^C-NMR (75 MHz, DMSO-d_6_) δ ppm: 33.7 (Me), 34.9 (CH_2_), 113.1, 114.1 (C-4, C-7), 114.3 (CN), 127.0, 127.2 (C-5, C-6), 130.1, 131.7 (C-4a, C-7a), 143.7 (C-2).

#### 3.1.2. General Procedure for the Synthesis of Pyrroles **17a**–**f**

Benzimidazolium bromide **15** (2 mmol) and the appropriate acetylenic dipolarophile (2.5 mmol) were refluxed with stirring in 1,2-epoxybutane (15 mL) for ca. 48 h. The solvent was evaporated under vacuum, and the residue was purified by column chromatography [Merck alumina (70–230 mesh), CH_2_Cl_2_].

*4-Benzoyl-1-[2-(methylamino)phenyl]-2-cyanopyrrole* (**17a**).

The compound was purified by crystallization from ethanol as colorless crystals with mp 144–145 °C; Yield 49%. Anal. (%) Calcd. for C_19_H_15_N_3_O (301.34 g/mol): C, 75.73; H, 5.02; N, 13.94. Found 75.98; H, 5.41; N, 14.24. IR (ATR solid, ν cm^−1^): 1637 cm^−1^ (ν_C=O_), 2220 cm^−1^ (ν_C≡N_), 3123 cm^−1^ (ν_CH_), 3341 cm^−1^ (ν_NH_); ^1^H-NMR (300 MHz, CDCl_3_) δ ppm: 2.83 (d, 3H, *J* = 4.7 Hz, **Me**NH), 3.55 (q, 1H, *J* = 4.7 Hz, NH), 6.77–6.82 (m, 2H, H-3′, H-5′, *N*-aryl), 7.17 (dd, 1H, *J* = 8.0, 1.4 Hz, H-6′, *N*-aryl), 7.36–7.44 (m, 1H, H-4′, *N*-aryl), 7.45 (d, 1H, *J* = 1.6 Hz, H-3, pyrrole), 7.46–7.52 (m, 2H, meta-phenyl), 7.51 (d, 1H, *J* = 1.6 Hz, H-5, pyrrole), 7.56–7.62 (m, 1H, para-phenyl), 7.83–7.86 (m, 2H, ortho-phenyl); ^13^C-NMR (75 MHz, CDCl_3_) δ ppm: 30.3 (**Me**NH), 107.1, 122.6 (2C, quaternary), 111.7 (C-3′), 111.9 (CN), 116.8 (C-5′), 122.4 (C-3), 125.7 (C-4), 127.7 (C-6′), 128.6, 129.0, 131.6, 138.4 (6 C, phenyl), 132.5 (C-4′), 132.8 (C-5), 144.7 (C-1′), 188.4 (CO).

*Methyl 1-[2-(methylamino)phenyl]-2-cyanopyrrole-4-carboxylate* (**17b**).

The compound was purified by crystallization from ethanol as colorless crystals with mp 140–142 °C; Yield 51%. Anal. (%) Calcd. for C_14_H_13_N_3_O_2_ (255.27 g/mol): C, 65.87; H, 5.13; N, 16.46. Found C, 66.12; H, 5.28; N, 16.67. IR (ATR solid, ν cm^−1^): 1705 cm^−1^ (ν_C=O_); 2226 cm^−1^ (ν_C≡N_); 3134 cm^−1^ (ν_CH_); 3403 cm^−1^ (ν_NH_); ^1^H-NMR (300 MHz, CDCl_3_) δ ppm: 2.75 (d, 3H, *J* = 4.9 Hz, **Me**NH), 3.42 (q, 1H, *J* = 4.9 Hz, NH), 3.77 (s, 3H, OMe), 6.69–6.73 (m, 2H, H-3′, H-5′, *N*-aryl), 7.06 (dd, 1H, *J* = 8.0, 1.4 Hz, H-6′, *N*-aryl), 7.29 (d, 1H, *J* = 1.6 Hz, H-3, pyrrole), 7.30–7.34 (m, 1H, H-4′, *N*-aryl), 7.45 (d, 1H, *J* = 1.6 Hz, H-5, pyrrole); ^13^C-NMR (75 MHz, CDCl_3_) δ ppm: 30.2 (**Me**NH), 51.8 (MeO), 107.1, 118.0, 122.4 (3C, quaternary), 111.6 (C-3′), 112.0 (CN), 116.7 (C-5′), 121.7 (C-3), 127.7 (C-6′), 131.5 (C-4′), 131.8 (C-5), 144.4 (C-1′), 163.3 (COO).

*Ethyl 1-[2-(methylamino)phenyl]-2-cyanopyrrole-4-carboxylate* (**17c**).

The compound was purified by crystallization from 2-propanol as colorless crystals with mp 145–148 °C; Yield 46%. Anal. (%) Calcd. for C_15_H_15_N_3_O_2_ (269.30 g/mol): C, 66.90; H, 5.61; N, 15.60. Found C, 67.22; H, 5.89; N, 15.87. IR (ATR solid, ν cm^−1^): 1699 cm^−1^ (ν_C=O_); 2223 cm^−1^ (ν_C≡N_); 3131 cm^−1^ (ν_CH_); 3410 cm^−1^ (ν_NH_); ^1^H-NMR (300 MHz, CDCl_3_) δ ppm: 1.28 (t, 3H, *J* = 7.1 Hz, **Me**CH_2_), 2.75 (d, 3H, *J* = 4.9 Hz, **Me**NH), 3.45 (q, 1H, *J* = 4.9 Hz, NH), 4.24 (q, 2H, *J* = 7.1 Hz, CH_2_O), 6.68–6.80 (m, 2H, H-3′, H-5′, *N*-aryl); 7.06 (dd, 1H, *J* = 8.0, 1.4 Hz, H-6′, *N*-aryl), 7.29–7.34 (m, 1H, H-4′, *N*-aryl), 7.31 (d, 1H, *J* =1.6 Hz, H-3, pyrrole); 7.45 (d, 1H, *J* = 1.6 Hz, H-5, pyrrole); ^13^C-NMR (75 MHz, CDCl_3_) δ ppm: 14.4 (**Me**CH_2_), 30.2 (**Me**NH), 60.6 (CH_2_O), 107.0, 118.5, 122.5 (3C, quaternary), 111.5 (C-3′), 112.0 (CN), 116.7 (C-5′), 121.7 (C-3), 127.7 (C-6′), 131.4 (C-4′), 131.7 (C-5), 144.7 (C-1′), 162.8 (COO).

*Isopropyl 1-[2-(methylamino)phenyl]-2-cyanopyrrole-4-carboxylate* (**17d**).

The compound was purified by crystallization from 2-propanol as colorless crystals with mp 154–156 °C; Yield 45%. Anal. (%) Calcd. for C_16_H_17_N_3_O_2_ (283.33 g/mol): C, 67.83; H, 6.05; N, 14.83. Found C, 68.17; H, 6.41; N, 15.11. IR (ATR solid, ν cm^−1^): 1699 cm^−1^ (ν_C=O_); 2228 cm^−1^ (ν_C≡N_); 3151 cm^−1^ (ν_CH_); 3395 cm^−1^ (ν_NH_); ^1^H-NMR (300 MHz, CDCl_3_) δ ppm: 1.33 (d, 6H, *J* = 6.1 Hz, **Me_2_**CH), 2.83 (s, 3H, **Me**NH), 3.42 (bs, 1H, NH), 5.20 (spt, 1H, *J* = 6.1 Hz, Me_2_**CH**), 6.76–6.80 (m, 2H, H-3′, H-5′, *N*-aryl), 7.12 (dd, 1H, *J* = 8.0, 1.4 Hz, H-6′, *N*-aryl), 7.35–7.41 (m, 1H, H-4′, *N*-aryl), 7.38 (d, 1H, *J* = 1.6 Hz, H-3, pyrrole), 7.49 (d, 1H, *J* = 1.6 Hz, H-5, pyrrole); ^13^C-NMR (75 MHz, CDCl_3_) δ ppm: 21.2 (**Me**_2_CH), 30.2 (**Me**NH), 68.0 (CH), 107.0, 118.9, 122.5 (3C, quaternary), 111.5 (C-3′), 112.0 (CN), 116.7 (C-5′), 121.6 (C-3), 127.6 (C-6′), 131.4 (C-4′), 131.6 (C-5), 144.6 (C-1′), 162.3 (COO).

*Dimethyl 1-[2-(methylamino)phenyl]-2-cyanopyrrole-3,4-dicarboxylate* (**17e**).

The compound was purified by crystallization from ethanol as colorless crystals with mp 185–187 °C; Yield 60%. Anal. (%) Calcd. for C_16_H_15_N_3_O_4_ (313.31 g/mol): C, 61.34; H, 4.83; N, 13.41. Found C, 61.63; H, 5.12; N, 13.66. IR (ATR solid, ν cm^−1^): 1752 cm^−1^ (ν_C=O_); 2232 cm^−1^ (ν_C≡N_); 3151 cm^−1^ (ν_CH_); 3382 cm^−1^ (ν_NH_); ^1^H-NMR (300 MHz, CDCl_3_) δ ppm: 2.76 (s, 3H, **Me**NH), 3.40 (bs, 1H, NH), 3.79 (s, 3H, MeO), 3.89 (s, 3H, MeO), 6.69–6.74 (m, 2H, H-3′, H-5′, *N*-aryl), 7.05 (dd, 1H, *J* = 8.0, 1.4 Hz, H-6′, *N*-aryl), 7.31–7.35 (m, 1H, H-4′, *N*-aryl), 7.36 (s, 1H, H-5, pyrrole); ^13^C-NMR (75 MHz, CDCl_3_) δ ppm: 30.3 (**Me**NH), 52.3, 52.8 (2MeO), 109.9, 117.8, 121.8, 125.8, 144.4 (5C, quaternary); 110.5 (CN), 111.9, 117.0, 127.7, 132.0, 132.2 (5C, tertiary), 161.8, 162.3 (2COO).

*Diethyl 1-[2-(methylamino)phenyl]-2-cyanopyrrole-3,4-dicarboxylate* (**17f**).

The compound was purified by crystallization from ethanol as colorless crystals with mp 134–136 °C; Yield 49%. Anal. (%) Calcd. for C_18_H_19_N_3_O_4_ (341.36 g/mol): C, 63.33; H, 5.61; N, 12.31. Found C, 63.71; H, 5.92; N, 12.54. IR (ATR solid, ν cm^−1^): 1715 cm^−1^, 1745 cm^−1^ (ν_C=O_); 2229 cm^−1^ (ν_C≡N_); 3394 cm^−1^ (ν_CH_); 3421 cm^−1^ (ν_NH_); ^1^H-NMR (300 MHz, CDCl_3_) δ ppm: 1.27 (t, 3H, *J* = 7.1 Hz, **Me**CH_2_), 1.34 (t, 3H, *J* = 7.1 Hz, **Me**CH_2_), 2.77 (s, 3H, **Me**NH), 3.38 (bs, 1H, NH), 4.25 (q, 2H, *J* = 7.1 Hz, CH_2_O), 4.36 (q, 2H, *J* = 7.1 Hz, CH_2_O); 6.69–6.74 (m, 2H, H-3′, H-5′, *N*-aryl); 7.05 (dd, 1H, *J* = 8.0, 1.4 Hz, H-6′, *N*-aryl), 7.31–7.35 (m, 1H, H-4′, *N*-aryl), 7.36 (s, 1H, H-5, pyrrole); ^13^C-NMR (75 MHz, CDCl_3_) δ ppm: 14.2, 14.3 (2Me), 30.2 (**Me**NH), 61.2, 62.0 (2CH_2_O), 109.7, 118.1, 121.9, 126.1, 144.6 (5C, quaternary), 110.5 (CN), 111.8, 116.9, 127.7, 131.9 (5C, tertiary), 161.4, 161.9 (2COO).

### 3.2. Toxicity Evaluation

#### 3.2.1. Phytotoxicity Evaluation

The toxicity of the new compounds on the plant species *Triticum aestivum* L. was evaluated by the D. Gr. Constantinescu method (*Triticum* biotest). This method is based on evaluating the elongation of the wheat rootlets at defined time intervals in the presence of the solutions or suspensions of the tested compounds [50,51].

Wheat grains are germinated for a period of 24 h. The grains that have germinated and present similar size and morphology are placed concentrically in Linhart vessels previously lined with cotton wool and filter paper. Germination takes place for an additional 24 h in an environment with corresponding humidity, at a temperature of 25 °C and in the dark. Germinated wheat grains with a length of 1 cm are placed in Petri dishes with a diameter of 10 cm, at a distance of 1 cm between each other. For the two control samples (distilled water and 1% DMSO solution in water) and for each dilution of the tested compounds, 11 germinated grains were chosen (10 to determinate root elongation, and one for microscopic examination).

For each compound, five successive dilutions were made in 1% DMSO, with the concentrations varying between 10 μM and 1000 μM. The linear elongation of the main root was measured at intervals of 24 h, 48 h, and 72 h.

Staining with orcein in acetic acid was used for the microscopic examination [52]. The examination of changes that took place in the nucleus and cell wall was performed with a Nikon Labophot-2 light microscope (Nikon, Chiyoda-ku, Tokyo, Japan) using 40× and 100× objective lenses, the latter under immersion with cedarwood oil (Sigma-Aldrich St. Louis, MO, USA).

#### 3.2.2. Animal Toxicity Assay

##### Artemia Franciscana Toxicity Assay

Crustaceans of the species *Artemia franciscana* Kellogg were used to determine the toxicity of the new pyrrole derivatives on the animal cell. The test assesses how many of the crustacean nauplii die in 24 h and 48 h intervals from the contact with the solutions of the tested compounds [53,54,55,56,57].

The artificial marine solution was obtained by dissolving CoralMarine Grotech sea salt in distilled water, 33.4 g/L. The hatching of crustacean cysts took place in a marine solution, 48 h in advance, at a temperature of 25 °C, with continuous oxygenation. The experiment was performed in 4 × 6 well plates and each dilution was applied in triplicate. Two mL of each dilution and approximately 12–15 nauplii were placed in a well. The evolution of nauplii was observed at 24 h and 48 h intervals, following how many of them died. The concentrations of the tested compounds evaluated ranged from 60–1000 µM.

##### Daphnia Magna Toxicity Assay

*Daphnia magna* Straus culture was maintained at 25 °C, and a photoperiod of 16 h/8 h light/dark cycle in a Sanyo MLR-351H climatic chamber (Sanyo, San Diego, CA, USA). Each compound was tested in six concentrations ranging from 50 to 1000 µM, and two replicates/concentration. For each replicate, ten daphnids were used, and the testing was performed in 12 well tissue culture plates (Greiner Bio-One, Kremsmünster, Austria) at a final volume/well of 4 mL [58,59,60,61]. Nifedipine in concentrations 1–1000 µM was used as the positive control, and a 1% DMSO solution as a negative control. The concentrations were selected based on the solubility and a pre-screening assay. The lethality was recorded at 24 and 48 h of exposure, and the LC_50_ values and the 95% confidence intervals (95%CI) for LC_50_ values were calculated using the least square fit method.

### 3.3. Prediction of the Molecular Mechanism of Action and Compounds’ Toxicity

The virtual screening was performed using PASS, a software designed to evaluate the pharmacological potential of new synthesized compounds. The structures of the new pyrrole derivatives were inputted as SMILES and the results were analyzed if the Pa values were above the corresponding Pi values. The freely available program GUSAR was used to predict the LD_50_ values of the new compounds after oral and intravenous administration on rats [62].

### 3.4. Statistical Analyses

The statistical analyses for the Triticum biotest were performed in the computing and programming language R, v. 4.0.3 (1), under Rstudio, v. 1.1.456 (RStudio, Inc., Version 4.0.3 (1), Boston, MA, USA). We used a conventional mixed-effects model (R package “lme4” [63]) and a robust mixed-effects model (R package “robustlmm” [64]) on measurements from all three days, treating the compounds and concentration as fixed effects and day of measurement as a random effect. Because evaluating the significance of multi-level regression is complex and controversial, besides estimating *p*-values using the Satterthwaite approximation (R package “lmerTest” [65]), we also used a more intuitive approach: ordinary least squares (OLS) and robust linear models („car“ [66], „robust“ [67] and „robustbase“ [68] R packages) were employed to assess the influence of difference compounds and concentrations (treated as factors) on root length, based on second day measurements (as the most representatives). Base R functions and a variety of packages („car”, „MASS” [69], „gvlma” [70]) were used for regression diagnostics. For effect size evaluation, the standardized regression coefficients (computed with the „lm.beta” [71] R package) were utilized. Violin plots were generated using the „ggplot” [72] R package. All calculations of *Daphnia magna* bioassay were performed using GraphPad Prism software (GraphPad Software, Inc., version 5.01, La Jolla, CA, USA).

## 4. Conclusions

A new efficient method for the synthesis of highly substituted pyrroles was achieved starting from 1-methyl-3-cyanomethyl-benzimidazolium bromide and acetylenic dipolarophiles. The new compounds from the pyrrole class were obtained by a two stage simple procedure implying the preparation of a benzimidazolium bromide and its one-pot reaction with symmetrical and non-symmetrical activated alkynes in 1,2-epoxybutane as the reaction medium. The new compounds were structurally characterized by IR, NMR, and X-ray single crystal diffraction of a representative compound.

The toxicity assessment of the compounds on the plant cells of *Triticum aestivum* L. (macroscopically and microscopically) and animal cells of crustacean *Artemia franciscana* Kellogg did not record any acute toxic (on *Triticum*) or lethal (on *Artemia* nauplii) effect, while the *Daphnia magna* bioassay and the PASS analysis results are encouraging for future research regarding the biological activity. Our findings are therefore suggestive of low acute toxicity and justify more research with respect to their long-term toxicity and their potential pharmacological applications.

## Figures and Tables

**Table 1:** Crystal data for compound **17c.**

Chemical Formula	C_15_H_15_N_3_O_2_
**Mr**	269.30
**Crystal system**	monoclinic
**Space group**	*P*2_1_/*c*
**Temperature (K)**	180
***a*, *b*, *c* (Å)**	11.3092(4), 11.6261(4), 10.8025(4)
***β*** **(** **°** **)**	96.930(4)
***V*** **(Å^3^)**	1409.96(10)
**Z**	4
**ρ_calcd_ (g cm^−3^)**	1.269
**Crystal size (mm)**	0.30 × 0.20 × 0.15
**μ (MoKα) (mm^−1^)**	0.087
**2*Θ* range**	3.628 to 58.318
**Reflections collected**	8712
**Independent reflections**	3261
***R*** **_int_**	0.0280
**No. of parameters**	183
**No. of restraints**	0
***R*** **_1obs_**	0.0477
***wR*** **_2obs_**	0.1008
***R*** **_1all_**	0.0703
***wR*** **_2al_** **l**	0.1137
**GOF**	1.037
**F(000)**	568
**completeness *Θ*_max_**	99.99%
**largest diff. peak/hole (e Å^–3^)**	0.15/−0.25
**CCDC No.**	2007999

**Table 2:** *Daphnia magna* bioassay: 24 h and 48 h values, 95% confidence.

Compound	LC ^1^_5024h_ (µM)	95% CI ^2^_24h_ (µM)	LC_5048h_ (µM)	95% CI_48h_ (µM)
**13**	259.9	222.4–303.7	136.0	110.2–167.9
**17a**	179.6	127.6–252.9	64.8	47.77–87.86
**17b**	121.3	89.7–164.0	ND *	13.27–43.19
**17c**	ND *	3.0–134.2	ND *	ND *
**17d**	889.6	850.7–930.2	410.4	ND **
**17e**	970.6	892.7–1055	496.2	408.9–602.1
**17f**	149.6	119.0–188.1	ND *	ND *
**Nifedipine**	ND *	ND *	8.3	7.1–9.7

^1^ LC50—50% lethal concentration; ^2^ 95% CI—95% confidence interval; ND not determined due to lethality values; * the compound induced lethality higher than 75% at all concentrations; ** 95%CI is too wide to be determined.

**Table 3:** The PASS predicted probability of the new pyrrole derivatives to be active (Pa) related to the calcium channel blockers.

Activity	17a	17b	17c	17d	17e	17f	Nifedipine
**Anti-ischemic cerebral**	0.43	0.53	0.59	0.67	0.38	0.46	0.65
**Anti-ischemic**	0.28	0.36	0.35	0.41	0.34	0.33	0.87
**Antianginal**	-	0.44	0.48	0.55	0.38	0.43	0.81
**Calcium channel (voltage-sensitive) activator**	0.36	0.45	0.43	-	0.53	0.51	0.68
**Cardiotonic**	0.33	0.38	0.39	0.41	0.45	0.46	0.41
**Calcium channel blocker**	-	0.29	0.34	0.32	0.28	0.32	0.72
**Antihypertensive**	-	0.30	0.32	0.39	0.20	0.23	0.97

**Table 4:** The PASS predicted probability of the new pyrrole derivatives to be active (Pa) as anti-arthritic drugs.

Activity	17a	17b	17c	17d	17e	17f
**Anti-arthritic**	0.67	0.61	0.58	0.51	-	-
**Interleukin antagonist**	0.63	0.54	0.50	0.48	-	-
**Interleukin 2 antagonist**	0.48	0.43	0.38	0.36	-	-
**Rheumatoid arthritis treatment**	0.40	0.46	0.46	0.37	0.36	0.37

**Table 5:** The predicted toxicity of compounds **17a**–**f**, nifedipine, and the modified structures **18a**–**d** expressed as median lethal doses (LD_50_) on rats using the GUSAR application.

Compound	Oral LD_50_ (mg/kg)	Oral LD_50_ Class	IV LD_50_ (mg/kg)	IV LD_50_ Class
**17a**	1984	4	65.75	4
**17b**	1412 *	4 *	71.56	4
**17c**	981	4	67.30	4
**17d**	809.8 *	4 *	40.72	4
**17e**	1735 *	4 *	120.60	4
**17f**	914.6	4	85.54	4
**nifedipine**	1225	4	57.46	4
**18a**	513.6 *	4 *	23.99	3
**18b**	1792	4	69.14	4
**18c**	877.6 *	4	46.77	4
**18d**	506.4	4	40.00	3

* The compound was out of the applicability domain of the model.

## Data Availability

The datasets used and/or analyzed during the current study are available from the corresponding author on reasonable request.

## Sample Availability

Samples of the compounds **17a**–**17**f are available from the authors.
